# A Treatise on the Practice of Medicine

**Published:** 1846-06

**Authors:** 


ARTICLE IX.

*A Treatise on the Practice of Medicine.* By JOHN EBERLE, Professor of the Theory and Practice of Medicine in Jefferson Medical College, etc. etc., with notes and additions by George McClellan, M. D., in two volumes. Sixth edition. Philadelphia: Grigg & Elliott. 1845. pp. 569, 565. (From the Publishers.)

It is only necessary to announce this new edition of Dr. Eberle's work, edited by his friend and former colleague Dr. McClellan. "In sound medical learning," says the editor, "in judicious criticism, and discriminating tact, our author scarcely had his superior." The value of the work is greatly enhanced by the notes added to this edition, and these will be highly prized by all who know how to estimate Dr. McClellan's opinions on all practical points.

We ought, perhaps, to add, as an apology for the late appearance of this notice, that the copy sent some months since, only lately came into our possession. D. B.

